# Influence of Aging on Bioaccumulation and Toxicity of Copper Oxide Nanoparticles and Dissolved Copper in the Sediment-Dwelling Oligochaete *Tubifex tubifex*: A Long-Term Study Using a Stable Copper Isotope

**DOI:** 10.3389/ftox.2021.737158

**Published:** 2021-10-01

**Authors:** Amalie Thit, Monica Hamann Sandgaard, Joachim Sturve, Catherine Mouneyrac, Anders Baun, Henriette Selck

**Affiliations:** ^1^ Department of Science and Environment, Roskilde University, Roskilde, Denmark; ^2^ Department of Biological and Environmental Sciences, University of Gothenburg, Gothenburg, Sweden; ^3^ Faculty of Sciences, BIOSSE, Université Catholique de L’Ouest, Angers, France; ^4^ Department of Environmental Engineering, Technical University of Denmark, Kongens Lyngby, Denmark

**Keywords:** metal, nanomaterial, bioavailability, effect, aging, freshwater, sediment, transformation

## Abstract

For engineered metal nanoparticles (NPs), such as copper oxide (CuO) NPs, the sediment is recognized as a major compartment for NP accumulation. Sediment-dwelling organisms, such as the worm *Tubifex tubifex*, will be at particular risk of metal and metal NP exposure. However, a range of complex transformation processes in the sediment affects NP bioavailability and toxicity as the contamination ages. The objective of this study was to examine bioaccumulation and adverse effects of CuO NPs in *T. tubifex* compared to dissolved Cu (administered as CuCl_2_) and the influence of aging of spiked sediment. This was done in a 28-day exposure experiment with *T. tubifex* incubated in clean sediment or freshly spiked sediment with different concentrations of dissolved Cu (up to 230 μg g^−1^ dw) or CuO NPs (up to 40 μg g^−1^ dw). The experiment was repeated with the same sediments after it had been aged for 2 years. To obtain a distinct isotopic signature compared to background Cu, both Cu forms were based on the stable isotope ^65^Cu (>99%). The 28-day exposure to sediment-associated dissolved ^65^Cu and ^65^CuO NPs resulted in a clear concentration-dependent increase in the *T. tubifex* ^65^Cu body burden. However, despite the elevated ^65^Cu body burdens in exposed worms, limited adverse effects were observed in either of the two experiments (e.g., above 80% survival in all treatments, low or no effects on the growth rate, feeding rate, and reproduction). Organisms exposed to aged sediments had lower body burdens of ^65^Cu than those exposed to freshly spiked sediments and we suggest that aging decreases the bioavailability of both ^65^Cu forms. In this study, the use of a stable isotope made it possible to use environmentally realistic Cu concentrations and, at the same time, differentiate between newly accumulated ^65^Cu and background Cu in experimental samples despite the high background Cu concentrations in sediment and *T. tubifex* tissue. Realistic exposure concentrations and aging of NPs should preferably be included in future studies to increase environmental realism to accurately predict the environmental risk of metal NPs.

## Introduction

Engineered metal nanoparticles (NPs), such as copper oxide (CuO) NPs, have unique properties as a result of their small size (1–100 nm) and high surface-to-volume ratio compared to their larger counterparts (Nel et al., 2006). CuO NPs and other Cu-containing NPs have vast applications, including antifouling paint, bioactive coatings, cosmetics, electronics, health products, inks, lubricants, plastics, solar cells, and batteries (Nanotechproject, 2021; Cioffi et al., 2005; Park et al., 2007; Wang et al., 2008; Perelshtein et al., 2009; Ren et al., 2009; Anyaogu et al., 2008). Therefore, there has been a dramatic increase in the use of Cu-containing particles over the past decades. As the production of CuO NPs increases the release of these particles *via* wastewater and agricultural runoff, release from weathered surfaces treated with NP-coatings and antifouling paints (Lammel et al., 2020) will likely cause increased exposure in the aquatic environment. In 2010, the annual production of Cu NPs was estimated to 200 tons year^−1^ and the release of these particles was estimated to 11 tons year^−1^ (Keller et al., 2013).

Aquatic sediments have been recognized as a major compartment for metal NP accumulation (Praetorius et al., 2012; Thit et al., 2015a; Cross et al., 2015). For example, the use of CuO NP-containing wood coatings in Europe alone could increase sediment concentration of CuO NPs by about 400 ng kg^−1^ annually (Caballero-Guzman and Nowack, 2018). According to Garner et al. (2017), CuO NP accumulation in the sediment compartment may lead to concentrations in the ng to μg kg^−1^ range (Garner et al., 2017). Due to this inevitable increase in sediment CuO NP concentration, sediment-dwelling, and especially deposit-feeding organisms such as the worm *Tubifex tubifex,* will be at particular risk of metal and metal NP exposure (Ozoh, 1992; Selck et al., 1998; Luoma and Rainbow, 2008; Croteau et al., 2011a; Burton, 2013; Thit et al., 2015a). In the few studies that have been conducted with sediment exposures, toxicity and bioaccumulation of CuO particles of varying sizes and shapes have been reported for sediment-dwelling snails, mussels, and worms (Gomes et al., 2012; Pang et al., 2012; Buffet et al., 2013; Pang et al., 2013; Ramskov et al., 2014; Thit et al., 2015a; Thit et al., 2015b; Thit et al., 2016; Thit and Selck, 2021).

In the aquatic environment, NPs will undergo a series of transformations. In the water column, CuO NPs will likely only marginally dissolve and rather agglomerate/aggregate, favoring their deposition onto sediment (Baun et al., 2008; Klaine et al., 2008; Quik et al., 2012; Thit et al., 2015a; Keller et al., 2017). In aquatic sediments, a range of transformation processes will occur, but the complex sediment matrix makes it very difficult to predict NP transformations in this compartment (Baun et al., 2017). Furthermore, increased contact time between metal NP and sediment (aging) may affect the bioavailability and toxicity of NPs in sediment. Compared to the literature on transformations occurring in the pelagic zone, little is known about these processes in sediment and the influence of aging on bioaccumulation and toxicity. Soil research has reported that prolonged contact time between NPs and soil generally decreased the toxicity of NPs (Jośko and Oleszczuk, 2013).

In the present study, the influence of aging on the toxicity and bioavailability of CuO NPs was studied using the freshwater oligochaete *T. tubifex* due to its important role in the ecosystem as a bioturbator greatly influencing the milieu (Matisoff et al., 1999; Anschutz et al., 2012; Thit et al., 2020), and as prey for a range of species, including fish, leading to potential trophic transfer of CuO NPs (Lammel et al., 2020). The objective of the study was to examine the potential bioaccumulation and adverse effects of CuO NPs compared to dissolved Cu and the influence of aging of spiked sediment on these processes. The study specifically addressed whether 1) bioaccumulation differed between Cu treatments (CuO NPs vs. dissolved Cu), 2) CuO NPs and dissolved Cu caused adverse effects in *T. tubifex* (in terms of altered reproduction, survival, growth, burrowing behavior, and feeding), and 3) aging of spiked sediment affected the bioaccumulation and adverse effects of CuO NPs and dissolved Cu. Since Cu is an essential metal and ubiquitously present in sediment and worm tissue, both Cu forms used in the present study were enriched in ^65^Cu (>99%) to obtain a distinct isotopic signature compared to background Cu in samples (Croteau et al., 2014; Zhang et al., 2019).

## Materials and Methods

### Test Chemicals and Nanoparticles

The ^65^CuCl_2_ stock solution was prepared by dissolving commercially purchased isotopically enriched ^65^CuCl_2_ (99.7% enrichment, Lot: 55-9, Trace Sciences International, United States) in Milli-Q water. Isotopically modified ^65^CuO NPs were synthesized as described previously (Lammel et al., 2021) using enriched ^65^Cu as a precursor (>99% purity, Trace Sciences International, United States) and CuO NPs from the same batch were used in the present study. Briefly, ^65^Cu was dissolved in a mixture of HCl and H_2_O_2_ followed by solvent removal on rotatory evaporator under vacuum at 90°C to obtain a powder of ^65^CuCl_2_. ^65^Cu NPs were synthesized by thermolysis of ^65^Cu-oleate obtained by reflux of a mixture of ^65^CuCl_2_ and sodium-oleate in water, ethanol, and hexane. Oxidation of the ^65^Cu NPs resulted in the formation of ^65^CuO NPs that was subsequently isolated *via* centrifugation and resuspended in 1 mmol/L sodium citrate solution. Characterization is presented in (Lammel et al., 2021). Briefly, primary particle size (20 nm), morphology (spherical), and surface chemistry (mixture phase of CuO, Cu_2_O, and Cu) were determined using TEM imaging (JEOL2100F at 200 kV) and X-ray diffraction (Bruker D8 advance diffractometer with a copper target).

### Test Organism: *T. Tubifex*

*T. tubifex* were originally purchased from Bonnies Dyrecenter (DK), where they have been stored at 4°C. The worms were acclimatized in the laboratory in glass aquaria with *T. tubifex* medium (media preparation described below) by gradually increasing temperature and storing at experimental conditions for 24 h (20°C in 16 h light and 8 h dark) before exposure. Aeration was provided using a pump, a silicon tube, and an aeration stone.

### Preparation of Artificial Freshwater

*Tubifex* medium (artificial freshwater) was prepared following OECD guideline 315. Briefly, 2 L stock solution was made by dissolving calcium chloride (11.76 g/L CaCl_2_-2H_2_O, 10035-04-8, Merck), magnesium sulfate (4.93 g/L MgSO_4_-7H_2_O, 10034-99-8, Merck), sodium bicarbonate (2.59 g/L, NaHCO_3_, 144-55-8, Merck), and potassium chloride (0.23 g/L KCl, 7447-40-7, Merck) in deionized water. Subsequently, the total volume was made up to 25 L with deionized water, aerated until oxygen saturation was achieved, and stored at 20°C.

### Sediment Preparation and Aging

Sediment was collected from a shallow area by Munkholmbroen in Isefjord, Roskilde, Denmark (55°40′25″N 11°48′44″E), in early spring 2019 by scraping off the top few centimeters of the sediment surface as described in (Ramskov et al., 2015). Subsequently, sediment was sieved at the site (<500 µm) using natural brackish water from the site to remove coarse debris and macrofauna. After settling (>72 h), the overlying water was carefully removed using a siphon and the sediment was frozen at −20°C to kill micro- and meiofauna. Then, the sediment was thawed and sieved to <63 µm with *T. tubifex* medium, and the overlying water was removed carefully after the sediment had settled after about a week. Sediment was rinsed by mixing with clean, aerated *T. tubifex* medium to obtain relevant salinity and homogenized thoroughly by hand mixing. Sediment was left to settle and the overlying water carefully removed, avoiding removal of organic material. Before spiking, a homogenous sediment slur was obtained using an immersion blender. The sediment was stored at 4°C in the dark until experimental use. Sediment aliquots were collected for the dry weight (dw): wet weight (ww) ratio determinations and organic matter content as described below.

The sediment was spiked (spring, 2019) by adding a known amount of the ^65^CuCl_2_ or ^65^CuO NP stock to a known amount of wet sediment. Four exposure concentrations per treatment were chosen to cover the range of environmentally relevant Cu concentrations and covering both low non-toxic and toxic concentrations (up to 230 µg ^65^Cu g^−1^ dw sediment). A lower concentration range was chosen for CuO NPs (up to 40 µg ^65^Cu g^−1^ dw sediment) than dissolved Cu as we expect the environmental concentrations to be lower for this Cu form. Uncontaminated control sediment was treated similarly, and the total volume of Milli-Q water added was equal among all treatments. Sediment (control and all Cu concentrations) was mixed thoroughly with a spoon and subsequently covered with parafilm and aluminum foil and placed on a shaking table for 72 h at room temperature (about 20°C) to obtain homogenous Cu distribution in the sediment. Sediment aliquots were collected to determine ^65^Cu concentration. Sediment was used immediately hereafter for experiments with freshly spiked sediment. The remaining spiked sediment was aged at 4°C in the dark for 2 years (23 months, until spring 2021).

The dw:ww ratio was determined for both freshly spiked (0.202 ± < 0.01; *n* = 6) and aged sediment (0.204 ± <0.01; *n* = 6) after drying sediment aliquots for 24 h at 105°C. The organic matter (OM) content (in freshly spiked sediment 18.1 ± 0.1%; *n* = 6 and aged sediment 18.6 ± 0.4%; *n* = 6) was determined after loss on ignition (>2 h at 550°C) on dry sediment aliquots.

### Experimental Setup

The setup was similar for both experiments (freshly spiked sediment and aged sediment). For all ten treatments, the respective sediment was transferred into each individual exposure container (25 ml scintillation vials: 2.5 cm in diameter and 5 cm in height). The amount of sediment per container corresponded to 1.5 g dw sediment (7.45 ± 0.03 g ww; *n* = 100, in experiment 1 with freshly spiked sediment; 7.37 ± 0.06 g ww; *n* = 100, in experiment 2 with aged sediment). The 9 ml aerated *Tubifex* media were gently added and exposure containers were covered with a lid and kept under experimental conditions overnight to allow sediment to settle. Healthy, sexually mature worms (with clitellum) of approximately similar size and age were carefully separated from the culture and pooled in groups of four. The worms were kept in six-well multi-well plates with aerated *Tubifex* media to empty their guts overnight. This depuration time was chosen in order to 1) be equal to depuration time after exposures (see below) to allow determination of worm growth rate during exposures and 2) to allow sufficient time to obtain fully empty guts (Thit and Selck, 2021). On the day of experimental initiation, 2/3 of the overlying water was decanted from the vials and replaced with newly aerated water and resuspended sediment was allowed to settle for 2 h. Immediately before exposure initiation, worms were weighed in groups of four (i.e., mean wet weight of each group: 15.71 ± 5.93 mg, *n* = 200 groups; corresponding to approximately 4 mg ww per worm in average).

In both experiments, exposures were initiated by adding four *T. tubifex* to each replicate exposure container with ten replicates per treatment and ten treatments in total (five concentrations for ^65^CuCl_2_ and five for ^65^CuO NPs), resulting in a total of 400 worms per experiment. Four worms per replicate were chosen to allow sufficient biomass for ^65^Cu body burden measurements. The time for each individual worm to burrow into the sediment was recorded for all treatments every minute for the first 10 min and then every 5–10 min for up to 2 h. Burrowing of worms was assessed as the number of worms (out of four) in each of the following categories: 1) worms in the overlying water, 2) partially buried (i.e., head only burrowed in the sediment), and 3) fully burrowed in sediment. Survival was noted, and fecal layer thickness was recorded at five selected days during exposures until day 27 of exposure. The fecal layer thickness was assessed following Thit et al. (2020). Briefly, the thickness of the fecal layer was measured at a predetermined place on the exposure container using an electronic caliper (Biltema, Dk), with 0.1 mm precision.

The test beakers were provided with air using silicone tubes with a needle connected to an aquarium pump. The beakers were covered with a plastic lid with holes for the air supply. Test beakers were placed in a climate cabinet at 20°C with a 16:8 h light-dark cycle. The test beakers were monitored every second day during the exposure period and evaporated water was replaced with DI water when needed. The pH of the overlying water was ∼7.5.

At experimental termination (i.e., day 28), approximately 7.5 ml of the overlying water, and subsequently fecal layer, was carefully removed from exposure containers using a pasture pipette, avoiding disturbing the sediment. Sediment was mixed carefully to get a homogenous slur and subsamples were retrieved for Cu concentration determination at T_end_ (making sure no worms or cocoons were in the sample) and frozen at -20°C. The remaining sediment (+worms and cocoons) was sieved through a 125 µm sieve and the sediment was washed with *Tubifex* media to expose surviving worms and cocoons and fecal matter. The adult worms were carefully retrieved from the sieve, washed, and transferred to multi-wells, where they were allowed to depurate overnight. The following day, the worms were weighed on an analytical scale to determine growth (weight change) and then frozen at −20°C. In the first experiment (i.e., with freshly spiked sediment), the cocoons were removed from the sieve and transferred to a multi-well with aerated *Tubifex* media and counted to examine fecundity. The cocoons were kept under experimental conditions for up to 3 months to examine the effects of treatment on hatching time and hatching efficiency. Every second day the number of hatched cocoons and the number of hatched worms were counted. Empty cocoons and newly hatched worms were removed from the wells. On the same census days, 2/3 of the water was removed and replaced with aerated *Tubifex* media. This process of collecting and counting cocoons, eggs, and newly born worms was very time-consuming and practically extremely challenging and was therefore only included in the first experiment (i.e., with freshly spiked sediment).

### Determining Concentrations of Background Cu and Newly Accumulated ^65^Cu

Cu concentrations in all samples (water, sediment, and worm tissue) were measured by inductively coupled plasma-mass spectrometry (ICP-MS, 7,900, Agilent) as previously described (Thit and Selck, 2021). Tissue and sediment samples were dried at 40°C for at least 48 h. All samples were subsequently digested according to ISO15587-2. Briefly, sediment and tissue samples were weighed into Teflon™ inserts (Milestone, Germany) and dissolved with 65% ultrapure nitric acid (HNO_3_) and Milli-Q water (1:1). Three Teflon™ inserts were placed in each Weflon™ vial (Milestone, Germany) containing 10 ml Milli-Q water and 2 ml 30% H_2_O_2_. Samples were heated in a microwave oven (START D Microwave Digestion System, Milestone, Germany) and subsequently transferred into volumetric flasks (resulting in 8% HNO_3_). Finally, Cu concentration in each sample was determined directly after digestion or after a short storage period (<24 h). A series of standard Cu solutions (8% HNO_3_) was used to calibrate Cu concentrations (six standards were selected from 0, 0.1, 2, 5, 10, 50, 100, 1,000 to 5,000 μg Cu L^−1^ to cover the range of expected Cu concentrations in the sample batch). During each analytical run, at least one of these standards was re-analyzed (approximately for every ten samples analyzed) to check for analytical drift and were all in agreement with expected Cu concentrations. To account for instrument drift and change in sensitivity, an internal standard (germanium ^74^Ge) was added to all samples. Samples were analyzed in duplicate (each analysis averaged 32 measurements) for the naturally occurring stable isotopes ^63^Cu and ^65^Cu by ICP-MS (Agilent). Samples were set to zero if their Cu concentrations inferred from ^63^Cu were higher than those of ^65^Cu.

Cu recovery was examined using a control solution with known Cu concentration (miljøkontrol 100 µg Cu L^−1^), certified mussel tissue (European reference materials, ERM®—CE278k; 5.98 μg Cu g^−1^ dw tissue), and certified freshwater sediment (RIZA, Trace metals WD CRM-CNS301-050; 44.2 μg Cu g^−1^ dw sediment), which were digested and analyzed together with different sample batches (at least one reference sample per batch). Results of control solution, certified tissue, and sediment were in good agreement with the certificate of analysis (control: 97.03 μg L^−1^ ± 3.2, *n* = 3; mussel: 5.47 ± 0.7 μg Cu g^−1^ dw tissue, *n* = 3; sediment: 40.1 ± 2.6 μg Cu g^−1^ dw sediment, *n* = 13, respectively). All equipment used for sample digestions was thoroughly acid-washed before use.

### Calculation of Concentrations of Newly Accumulated ^65^Cu

Concentrations of newly accumulated or added ^65^Cu (referred to as ^65^Cu in the following) were calculated based on ICP-MS measurements of the two naturally abundant stable isotopes, ^65^Cu and ^63^Cu (Croteau et al., 2004; Thit and Selck, 2021). Briefly, the relative abundance of the two isotopes in natural Cu samples in the absence of a spike (p^65^) was set to 0.309. Concentrations of newly accumulated ^65^Cu in the experimental organisms ([^65^Cu]_org_) were calculated as the product of p^65^ and the total Cu concentrations inferred by the ICP-MS software from ^65^Cu intensity ([T^65^Cu]):

[C65u]org = p65 ×[ TC65u]. (1)

The original load of tracer ([^65^Cu]org^0^) that occurred in each sample in the absence of a ^65^Cu spike was calculated as the product of p^65^ and the total Cu concentrations inferred from the intensity of the most abundant Cu isotope, ([T^63^Cu]):

[C65u]org0 = p65 × [TC63u]. (2)

The net tracer uptake (Δ[^65^Cu]_org_) was derived from the total Cu concentration inferred from ^65^Cu signal ([^65^Cu]_org_) minus the pre-existing concentration of tracer ([^65^Cu]_org_^0^):

Δ[C65u]org = [C65u]org − [C65u]org0 = p65 × ([TC65u] - [TC63u]). (3)

### Statistical Analysis

All statistical analyses were conducted using SYSTAT, version 13, and graphical data presentations were made using SigmaPlot for windows, version 14. (Systat Software, Inc., San Jose, CA, United States). All data are presented as mean ± one standard deviation (SD). Whether data fitted the assumptions for parametric analysis, i.e., normally distributed data with homogeneous variances, was tested using Kolmogorov–Smirnov and Levene’s test, respectively. One-way Analysis of Variance (one-way ANOVA) was conducted when data met the requirements for parametric tests. Tukey’s Honestly Significant Difference Test was used when significant differences were detected to determine significant pairwise differences between samples. When data (or transformed data) did not meet the requirements for parametric analysis, a nonparametric Kruskal–Wallis analysis on ranks was performed, followed by Conover–Inman Test for all pairwise comparisons. When comparing the two samples, a Student’s two-sample *t*-test was conducted. Differences were considered significant when *p* ≤ 0.05.

## Results

### Cu and ^65^Cu Concentrations in Sediment

The average background Cu concentration in the control sediment (sieved to <63 μm) was 72.5 ± 7.4 μg Cu g^−1^ dry weight (dw) sediment (*n* = 11). Note that the high background Cu concentration is a result of the small sediment grain size compared to, for example, the same sediment sieved to greater grain size (<125 μm; 23 µg Cu g^−1^ dw sediment) (Thit and Selck, 2021). As expected, the newly added ^65^Cu concentration was negligible (0.1 ± 0.1 μg ^65^Cu g^−1^ dry weight sediment; *n* = 11). Sediment ^65^Cu concentrations were measured immediately after spiking (T_0, freshly spiked_), after 28 days of exposure in freshly spiked sediment (T_end, freshly spiked_), at exposure initiation of experiments in aged sediment (T_0, aged_), and after 28 days in experiments with aged sediment (T_end, aged_). Measured sediment ^65^Cu concentrations increased with increasing nominal concentrations for experiments with both freshly spiked and aged sediment at both the beginning and end of exposure (Table 1). In the following, exposure ^65^Cu concentrations are reported as the mean measured concentrations at the initiation of exposures with fresh sediment (i.e., for ^65^CuCl_2_: 0, 18, 53, 112, 227 µg ^65^Cu g^−1^ dw sediment and for ^65^CuO NP: 0, 3, 8.9, 18, 36.5 µg ^65^Cu g^−1^ dw sediment). In general, there were limited statistically significant changes in exposure concentrations from the beginning to the end of exposures in either of the two experiments (see Supplementary Material, SI, for statistical comparison). Furthermore, ^65^Cu levels at the beginning of exposures with newly spiked and aged sediment in control sediment were not statistically significantly different from each other (*p* = 0.694 and 0.648 for ^65^CuCl_2_ and ^65^CuO NP, respectively). Sediment ^65^Cu concentrations in the majority of the treatments were slightly higher after aging than right after spiking (significant for most concentrations; see SI for further info).

**TABLE 1:** Sediment ^65^Cu concentrations from experiments with freshly spiked sediment and sediment aged for 2 years.

Compound	Nominal conc.	Freshly spiked sediment (µg ^65^Cu g^−1^ dw sed)		Two-year aged sediment (µg ^65^Cu g^−1^ dw sed)	
		T_0, fresh_	T_end, fresh_	T_0, aged_	T_end, aged_
^65^CuCl_2_	0	0.2 ± 0.2	0.1 ± 0.0	0.3 ± 0.2	0.4 ± 0.1
	18	18.1 ± 0.3	17.1 ± 2.7	21.2 ± 0.3	19.1 ± 4.3
	53	52.9 ± 1.8	49.0 ± 4.8	64.3 ± 0.6	56.7 ± 10.2
	112	112.1 ± 2.3	111.8 ± 1.9	129.2 ± 2.0	86.2 ± 32.8
	227	227.3 ± 6.1	232.4 ± 12.2	256.1 ± 1.4	203.6 ± 4.5
^65^CuO NP	0	0.1 ± 0.2	0.1 ± 0.0	0.2 ± 0.1	0.4 ± 0.2
	3	3.2 ± 0.2	0	3.4 ± 0.1	3.6 ± 0.7
	8.9	8.9 ± 0.1	7.7 ± 1.3	9.7 ± 0.2	8.2 ± 0.5
	18	17.9 ± 0.5	14.8 ± 1.1	19.9 ± 0.2	21.5 ± 1.8
	36.5	36.5 ± 6	31.9 ± 1.5	41.0 ± 0.5	39.8 ± 8.4

For the experiment with freshly spiked sediment, sediment samples were collected after spiking (i.e., at exposure initiation; T_0, fresh_), and after exposure termination (T_end, fresh_). For experiments with aged sediment, sediment samples were collected after aging (at exposure initiation; T_0, aged_) and at exposure termination (T_end, aged_). Concentrations are presented as mean ± SD; µg ^65^Cu g^−1^ dw sediment. *n* = 3 for all samples, except for T_0, aged_, where *n* = 2.

### Cu and ^65^Cu Concentrations in Overlying Water

Background Cu concentrations (total Cu) in overlying water of control sediment were 18.8 ± 5.3 μg L^−1^ (*n* = 8) for freshly spiked sediment and 12.0 ± 5.1 μg L^−1^ (*n* = 6) for aged sediment. ^65^Cu concentrations in overlying water (unfiltered samples containing dissolved Cu and Cu sorbed to colloids) collected at the termination of experiments with freshly spiked sediment and aged sediment from both ^65^CuCl_2_ and ^65^CuO NP exposures increased significantly with increasing sediment concentrations (all four *p-values* <0.001; Kruskal–Wallis; Table 2).

**TABLE 2:** Water ^65^Cu concentrations from experiments with freshly spiked sediment and sediment aged for 2 years.

Compound	Nominal conc.	Freshly spiked sediment (µg ^65^Cu L^−1^)	Two-year aged sediment (µg ^65^Cu L^−1^)
		T_end, fresh_	T_end, aged_
^65^CuCl_2_	0	0.1 ± 0.0	0.0 ± 0.0
	18	12.8 ± 4.1	4.1 ± 1.2
	53	32.9 ± 8.2	9.5 ± 2.3
	112	59.7 ± 5.9	19.0 ± 3.4
	227	73.3 ± 4.0	31.9 ± 10.7
^65^CuO NP	0	0.0 ± 0.0	0.0 ± 0.0
	3	2.9 ± 1.0	0.6 ± 0.5
	8.9	7.8 ± 1.8	0.7 ± 0.2
	18	12.6 ± 3.0	2.7 ± 1.0
	36.5	20.8 ± 4.8	5.5 ± 1.9

Samples were collected at exposure termination after 28 days (T_end, fresh_, T_end, aged_). Concentrations are presented as mean ± SD; µg ^65^Cu L^−1^, *n* = 5.

In controls (with unspiked control sediment), ^65^Cu concentrations in overlying water were close to zero and did not significantly differ between experiments with freshly spiked or aged sediment (*p* >0.05; two-sample *t*-test). However, ^65^Cu concentrations in overlying water at the end of exposures in freshly spiked sediment were significantly higher than those from aged sediment for both ^65^CuCl_2_ and ^65^CuO NP (all *p*-values <0.01; two-sample *t*-test).

### *T. tubifex* ^65^Cu Weight-Specific Body Burden

The background concentration of Cu (total Cu) in control worms was 30.3 ± 10.3 µg Cu g^−1^ dw tissue. As expected, the concentration of newly added ^65^Cu in *T. tubifex* from control treatments (i.e., sediment with no added ^65^Cu) was negligible at 0.2 ± 0.2 μg g^−1^ dw tissue (*n* = 20).

In both treatments, the added ^65^Cu was bioavailable and resulted in an increased ^65^Cu weight-specific body burden (WS-BB) in *T. tubifex* after 28 days of exposure. The WS-BB of worms exposed in sediment with ^65^CuCl_2_ in freshly spiked or aged sediment was significantly affected by ^65^Cu concentration (both *p* <0.001; Kruskal–Wallis). In freshly spiked sediment, the WS-BB of worms in all treatments was significantly different from control and from each other (all *p*-values <0.001; Conover–Inman). In aged sediment, the WS-BB differed significantly from the control for worms exposed at 227 µg ^65^Cu g^−1^ dw sediment and marginally at 112 µg ^65^Cu g^−1^ dw sediment (*p* = 0.018 and 0.052, respectively).

For ^65^CuO NPs, the WS-BB of worms in both freshly spiked and aged sediments differed significantly among concentrations (both *p-*values <0.001; Kruskal–Wallis). In freshly spiked sediment, the WS-BB of worms in all treatments was significantly higher than that in control and differed among concentrations (*p*-values <0.01 for all comparisons to control and *p*-values <0.05 for all comparisons between other treatments; Conover–Inman). In aged sediment, only the WS-BB of worms in the highest concentration, 36.5μg g^−1^ dw sediment, was significantly higher than that in control (*p* = 0.015).

**FIGURE 1:**
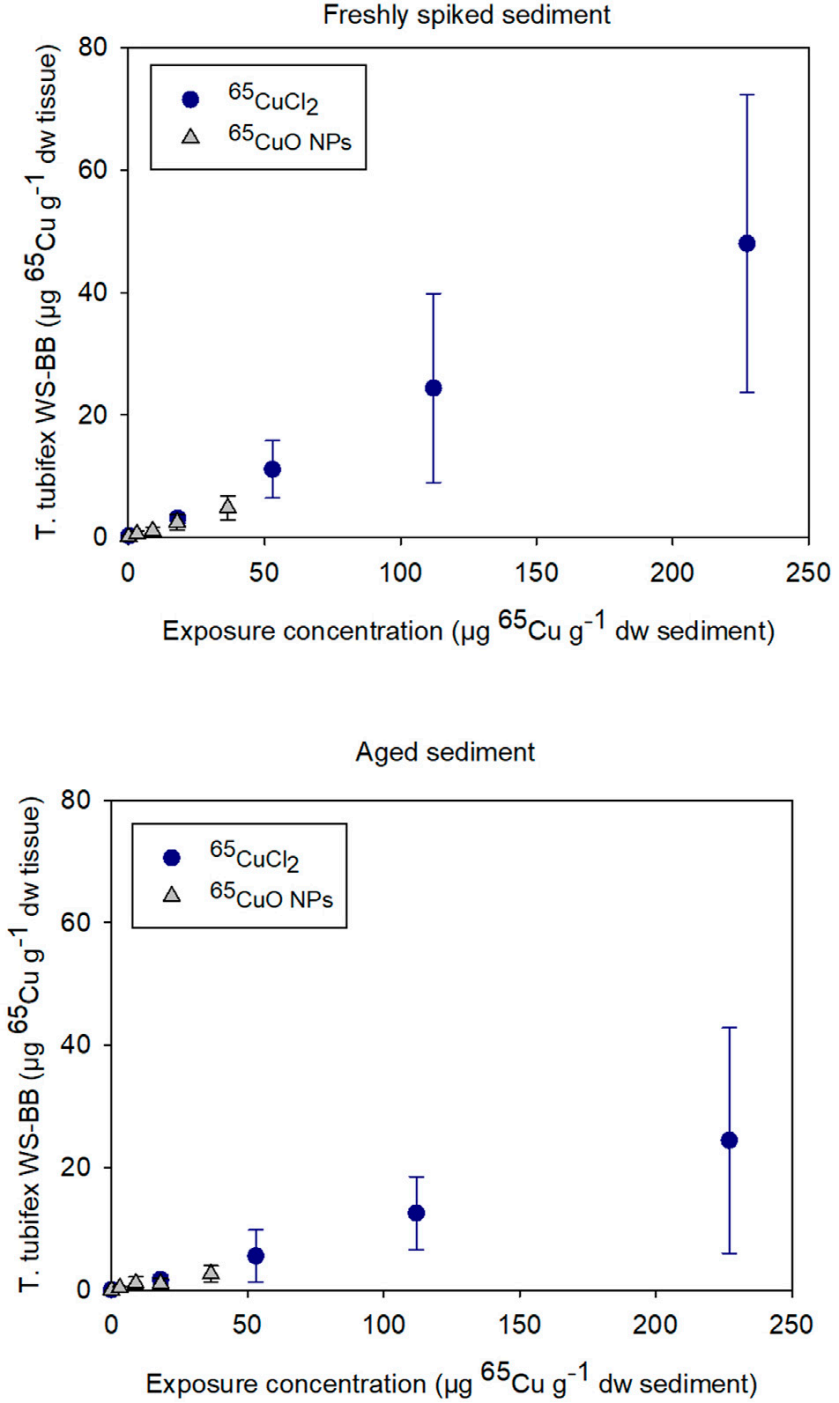
Weight-specific body burden (WS-BB) of newly accumulated ^65^Cu in *T. tubifex* after 28-day exposure to five concentrations of ^65^CuCl_2_ or ^65^CuO NPs in freshly spiked or 2-year aged sediment. WS-BB is presented as mean µg ^65^Cu g^−1^ dw tissue ±SD, *n* = 8–10.

As seen in Figure 1, bioaccumulation of ^65^Cu after exposure to ^65^CuCl_2_ and ^65^CuO NPs seemed to increase similarly with increasing sediment exposure concentration in both experiments with freshly spiked or aged sediment. The ^65^Cu WS-BB for organisms exposed to newly spiked sediment was higher than that for organisms exposed to aged sediment compared to worms exposed to similar ^65^Cu form and concentration.

For ^65^CuCl_2_, there was a significant difference between WS-BB in worms exposed to freshly spiked and aged sediment in sediments with 18, 53, and 227 µg ^65^Cu g^−1^ dw sediment (*p <*0.001, 0.013, and 0.029, respectively; two-sample *t*-test; Supplementary Figure S1). For control worms and those exposed to 112 µg ^65^Cu g^−1^ dw sediment, there was no significant difference (*p* > 0.05). For ^65^CuO NPs, the WS-BB of worms in freshly spiked sediment was significantly higher than that in aged sediment, when exposed to the two highest concentrations, 18 and 36.5 µg ^65^Cu g^−1^ dw sediment (*p* = 0.010 and 0.012, respectively; Two-sample t-test). Thus, the 28-day exposures to ^65^CuCl_2_ and ^65^CuO NPs in freshly spiked or aged sediment revealed that ^65^Cu accumulation in *T. tubifex* was influenced by ^65^Cu exposure concentration and aging but not on ^65^Cu form (Figure 1).

### Adverse Effects

#### Survival and Burrowing Activity of *T. Tubifex*

**FIGURE 2:**
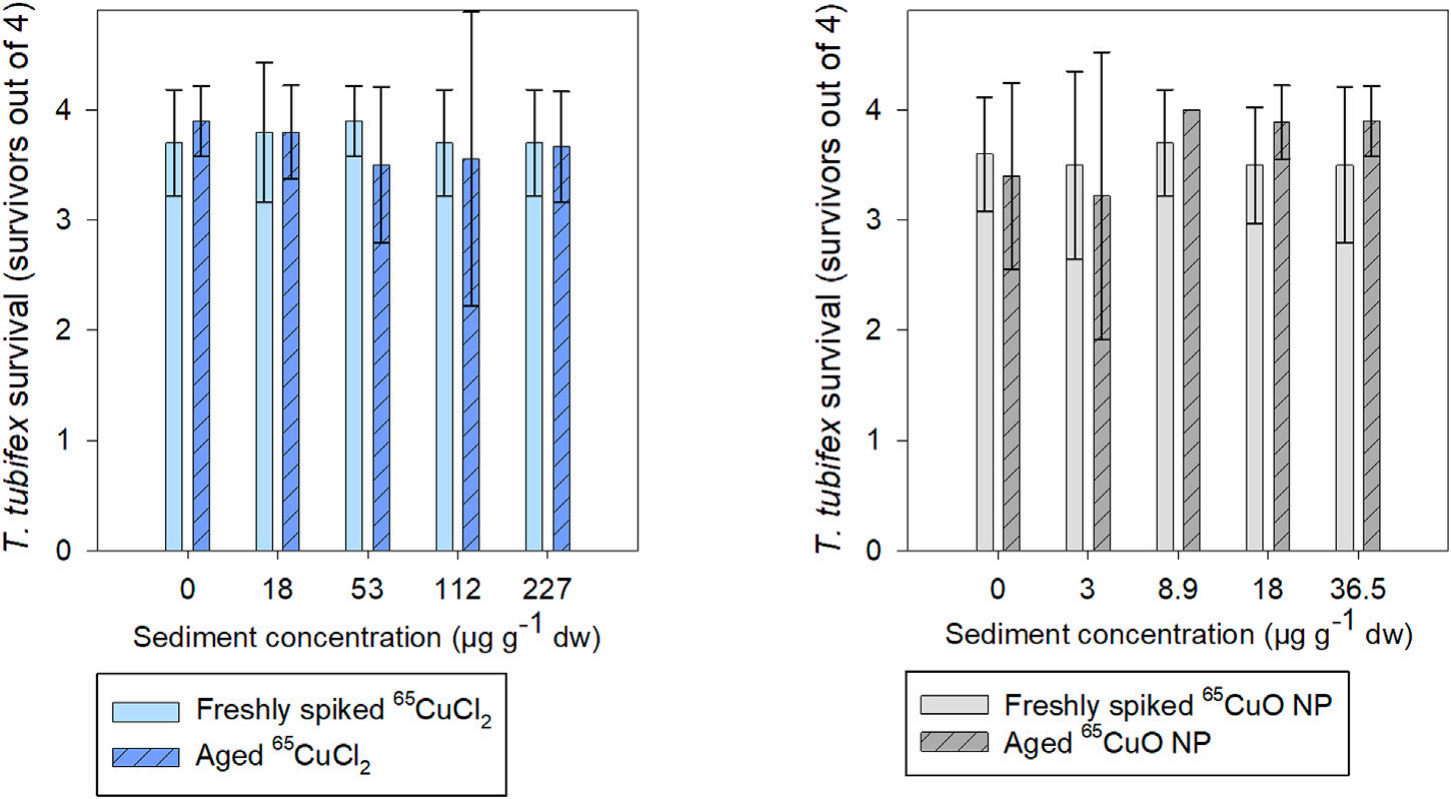
Number of surviving *T. tubifex* out of four per replicate after exposure in freshly spiked sediment (full bars) or aged sediment (scratched bars). Worms were exposed in sediment with ^65^CuCl_2_ (blue) or ^65^CuO NPs (gray) at five concentrations of ^65^CuCl_2_ or ^65^CuO NPs for 28 days. Bars represent the mean number of surviving worms ±SD, *n* = 10.

About 90% of *T. tubifex* survived during experiments with freshly spiked sediment and aged sediment, 88–98% in freshly spiked sediment, and 81–100% in aged sediment (Figure 2). No influence of ^65^Cu treatment, exposure concentration, or sediment aging on *T. tubifex* survival was detected during exposures.

**FIGURE 3:**
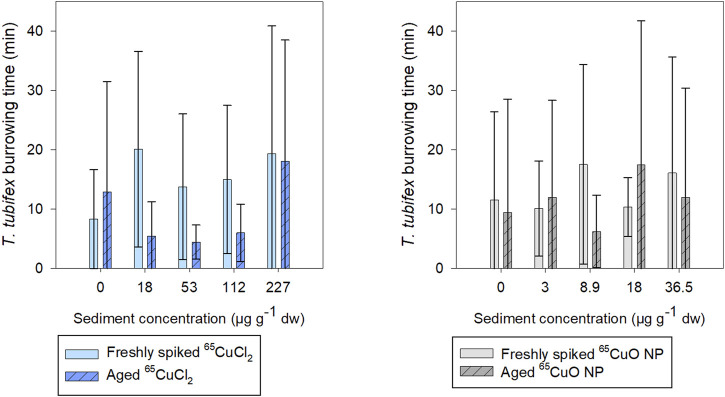
Time until *T. tubifex* was completely buried in sediment after initiation of exposure in freshly spiked sediment (full bars) or aged sediment (scratched bars). Worms were exposed in sediment with ^65^CuCl_2_ [blue, **(A)**] or ^65^CuO NPs [gray, **(B)**] at five concentrations. Bars represent the mean time for all four worms to be completely burrowed ±SD, *n* = 10.

As seen in Figure 3, the burrowing activity of *T. tubifex* in control sediments was not affected by the aging of sediment. There was no significant difference in the mean burrowing time in freshly spiked sediment (11 ± 18 min) and aged sediment (10 ± 12 min) (*p* = 0.814; two-sample *t*-test). Furthermore, in spiked sediments, there was no significant effect of ^65^Cu treatment, concentration, or sediment aging on burrowing time (freshly spiked sediment with ^65^CuCl_2_: *p* = 0.140; aged sediment with ^65^CuCl_2_: *p* = 0.592; freshly spiked sediment for both treatments: *p* = 0.192; freshly spiked sediment with ^65^CuO NP: *p* = 0.374; aged sediment with ^65^CuO NP: *p* = 0.568; aged sediment for both treatments: *p* = 0.283; Kruskal–Wallis). Thus, the 28-day exposures to ^65^CuCl_2_ and ^65^CuO NPs in freshly spiked or aged sediment did not result in any detectable influence of ^65^Cu form, ^65^Cu exposure concentration, or aging on *T. tubifex* survival and burrowing activity (Figure 2).

#### *T. Tubifex* Growth

**FIGURE 4:**
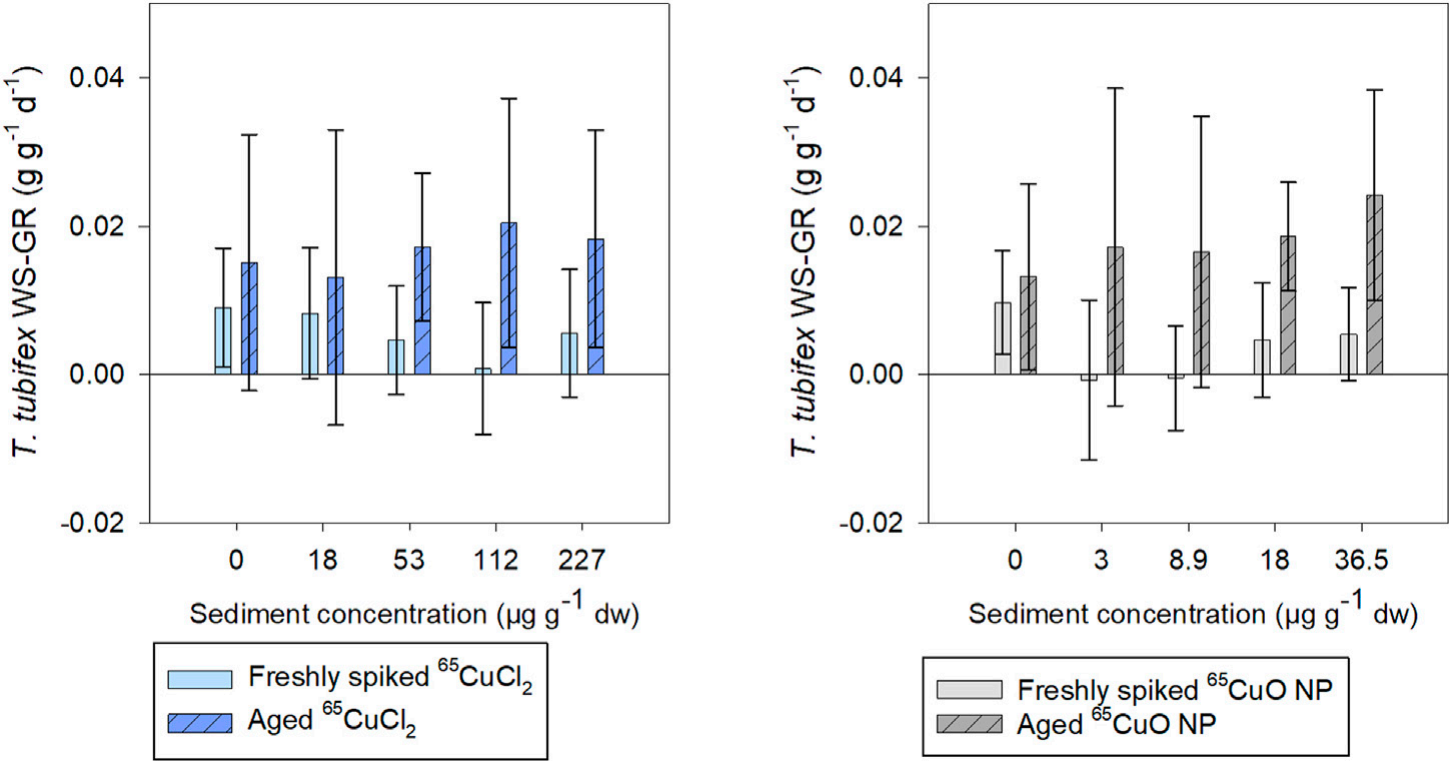
*T. tubifex* weight-specific growth rate (WS-GR) during exposure in freshly spiked sediment (full bars) or aged sediment (scratched bars). Worms were exposed in sediment with ^65^CuCl_2_ [blue, **(A)**] or ^65^CuO NPs [gray, **(B)**] at five different concentrations for 28 days. Bars represent the mean WS-GR ± SD, *n* = 10.

Weight-specific growth rate (WS-GR) of control worms was close to zero (as expected for sexually mature worms) during exposures in freshly spiked and aged sediment (Figure 4). Furthermore, there were no significant differences in WS-GR between the two control groups (^65^CuCl_2_ or ^65^CuO NPs) in either of the two experiments (*p* = 0.846 in freshly spiked sediment and 0.786 in aged sediment; two-sample *t*-test). In spiked sediments, there were no significant effects of ^65^Cu treatments or ^65^Cu concentration on WS-GR in neither freshly spiked nor aged sediment (in freshly spiked sediment: *p* = 0.251 for ^65^CuCl_2_ and *p* = 0.718 for ^65^CuO NPs; in aged sediment: *p* = 0.472 for ^65^CuCl_2_ and *p* = 0.108 for ^65^CuO NPs; ANOVA).

In experiments with freshly spiked sediment, worms were slightly bigger and had slightly lower growth rates than worms in aged sediment (0.009 and 0.014 g g^−1^ ww tissue d^−1^, respectively; *n* = 20 per sample), though not significantly different (*p* = 0.204; two-sample *t*-test).

WS-GR of worms from ^65^CuCl_2_ or ^65^CuO NP control treatments did not differ significantly between experiments with freshly spiked sediment and aged sediment (*p* = 0.328 and 0.448, respectively; two-sample *t*-test). However, the WS-GR of worms in freshly spiked sediment differed significantly from those exposed to aged sediment with ^65^CuCl_2_ for all treatments (18, 112, and 227; *p*-values = 0.030, 0.007, and 0.041, respectively) except at 53 µg ^65^Cu g^−1^ dw sediment (*p* = 0.233; two-sample *t*-test). For worms exposed to ^65^CuO NPs, WS-GR also differed between the two experiments (8.9, 18, and 36.5 μg g^−1^ dw sediment; *p*-values = 0.018, 0.001, and 0.002, respectively; two-sample *t*-test) except at the lowest exposure concentration of 3 µg ^65^Cu g^−1^ dw sediment (*p* = 0.057). Thus, *T. tubifex* weight-specific growth rate (WS-GR) was not affected by ^65^Cu form or exposure concentration in sediment but was affected by 2 years of aging of sediment with ^65^CuCl_2_ and ^65^CuO NPs.

#### *T. Tubifex* Feeding Rate: Egestion

**FIGURE 5:**
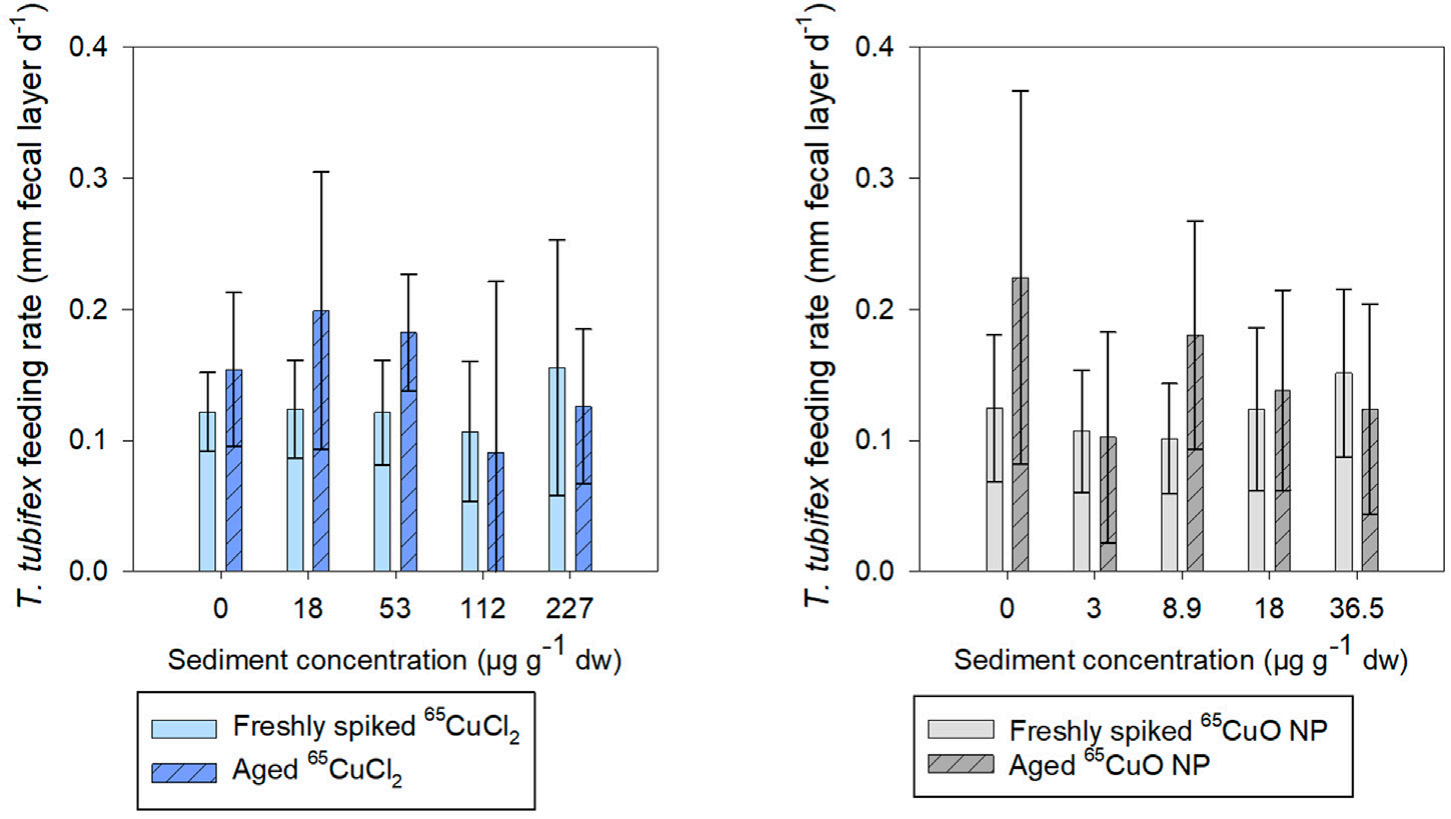
*T. tubifex* feeding rate measured as increase in fecal layer thickness (mm d^−1^) at day 27 of exposure in freshly spiked (full bars) or aged (schratched bars) sediment with ^65^CuCl_2_ [blue, **(A)**] or ^65^CuO NPs [gray, **(B)**] at five different concentrations. Fecal layer thickness was measured with calipers at a pre-destined place of the exposure vial. mean ± SD, *n* = 10.

It was found that fecal layer increased throughout exposures for all treatments in both freshly spiked and aged sediment indicating that worms were actively feeding throughout both experiments (Supplementary Figure S2 in SI). In freshly spiked sediment, there was a tendency for worms in the control treatments to produce slightly more fecal matter throughout exposures compared to worms exposed to ^65^CuCl_2_ (Supplementary Figure S2). However, after 27 days of exposure, there was no effect of ^65^Cu concentration on worm fecal matter production in freshly spiked or aged sediment with ^65^CuCl_2_ (*p* = 0.192 and 0.067, respectively; ANOVA) or ^65^CuO NP (*p* = 0.080 and 0.180, respectively; ANOVA) (Figure 5).

There was no influence of aging on the fecal matter production of control worms, and limited effects were observed on feeding of worms in sediments spiked with either ^65^Cu form. For worms exposed to ^65^CuCl_2_, there was no significant difference between fecal matter production in freshly spiked sediment and aged sediment (control, 18, 36.5, 112, and 227 µg ^65^Cu g^−1^ dw sediment; *p* = 0.142, 0.058, 0.722, and 0.422, respectively; two-sample t-test) except at 36.5 µg ^65^Cu g^−1^ dw sediment (*p* = 0.005).

For worms exposed to ^65^CuO NPs, there was no significant difference between fecal matter production in freshly spiked sediment and aged sediment (control, 3, 18, and 36.5 µg ^65^Cu g^−1^ dw sediment; *p* = 0.063, 0.881, 0.647, and 0.412, respectively; two-sample *t*-test) except at 8.9 µg ^65^Cu g^−1^ dw sediment (*p* = 0.022). Thus, limited influence of ^65^Cu treatment, exposure concentration, or aging on feeding rate was observed during exposures.

#### Reproductive Effects

**FIGURE 6:**
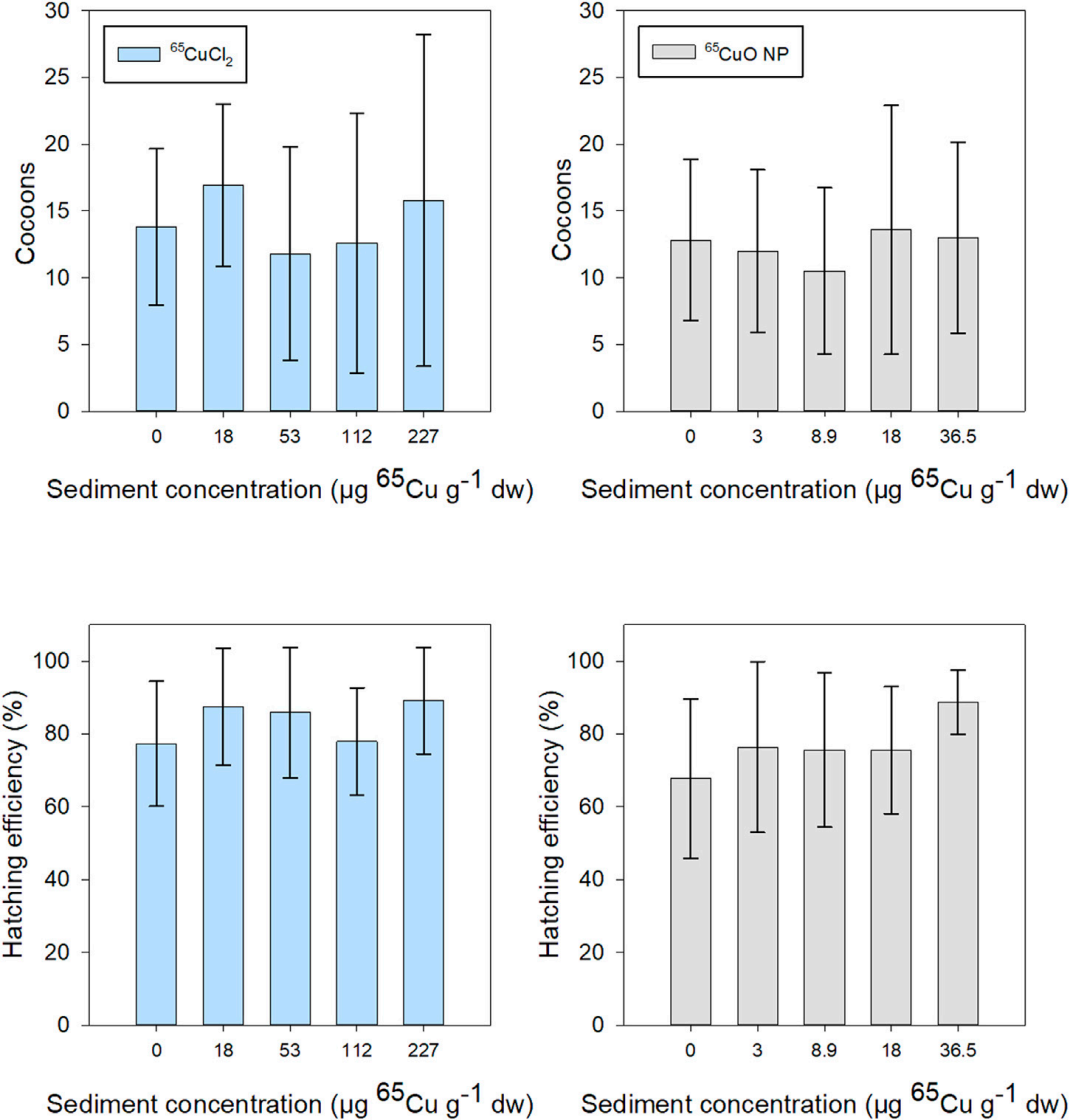
*T. tubifex* reproduction during exposures in freshly spiked sediment with ^65^CuCl_2_ (blue) or ^65^CuO NPs (gray) at five different concentrations. Reproductive output is presented as the mean ± SD number of cocoons at the end of exposure and hatchability (number of cocoons hatched, 2.5 months after exposure termination). *n* = 10.

The mean number of cocoons produced during 28 days of exposure in freshly spiked sediment was about 15 for *T. tubifex* in all treatments. Hatching efficiency after 2.5 months after exposure termination was generally about 75% and above 60% in all treatments. There was a tendency for increased hatchability with increasing exposure concentration in both ^65^Cu treatments. However, there was no significant effect of ^65^Cu sediment concentration on the number of cocoons or hatching efficiency for either ^65^CuCl_2_ (*p* = 0.532 and 0.223, respectively; Kruskal–Wallis) or ^65^CuO NPs (*p* = 0.929 and 0.301, respectively; Kruskal–Wallis) (Figure 6). Thus, limited influence ^65^Cu treatment or exposure concentration on *T. tubifex* reproduction was observed. Reproduction was only assessed for experiments in freshly spiked sediment and therefore no data are presented for reproduction in aged sediment.

## Discussion

### Exposure Characterization

In the present study, background Cu concentrations in overlying water of sediments were up to about 70 µg ^65^Cu L^−1^, which is in the range of naturally occurring Cu concentrations in freshwater (0.2–100 μg L^−1^) (Cole et al., 1984; Misra et al., 2012a). The sediment exposure concentrations (up to 230 µg ^65^Cu g^−1^ dw sediment for dissolved Cu, administered as ^65^CuCl_2_) were selected to reflect the range of Cu sediment concentrations occurring in uncontaminated (often 5–50 µg Cu g^−1^ dw sediment) to highly contaminated sites (mg Cu g^−1^ dw range). In the present study, background Cu concentrations in sediment sieved to 63 µm ( ~ 70 µg Cu g^−1^ sediment) also reflected naturally occurring background Cu concentrations. Though concentrations of CuO NPs in sediments are expected to increase considerably in the future (Caballero-Guzman and Nowack, 2018), the estimated sediment concentrations of CuO NPs are currently in the ng Cu g^−1^ range (Garner et al., 2017). Thus, lower exposure concentrations of up to 40 µg ^65^Cu g^−1^ dw sediment for ^65^CuO NPs were selected to increase environmental realism. Though these concentrations are still higher than the generally expected concentrations of CuO NPs in the environment, point sources may lead to hot spots with higher CuO NP concentrations. Exposures to CuO NPs will be chronic and aging should be considered when examining the risk of these NPs in the environment (Lowry et al., 2012).

Transformations of CuO NPs (as well as Cu administered as CuCl_2_) will inevitably occur in the environment, affecting bioavailability and toxicity. Compared to the literature on transformations occurring in the pelagic zone, little is known about transformations in sediment. The complex and heterogenic sediment matrix combined with the dynamic and stochastic nature of the environmental system makes it very difficult to predict these transformation processes in the sediment compartment (Lowry et al., 2012; Cross et al., 2015). However, aggregation/agglomeration, sorption on solids, adsorption of macromolecules, such as proteins, on the particle surface (referred to as corona formation), sulfidation, dissolution, and redox reactions in the environment are expected to be pronounced for metal oxide NPs (Lowry et al., 2012; Ma et al., 2014; Cross et al., 2015; Baun et al., 2017). During the 2 years of aging in the present study, transformations, such as reduction, have likely been pronounced due to the low oxygen levels present in the sediment. Though the solubility of CuO NPs in the aquatic environment (at neutral pH) has generally been reported to be low (below 3% of the original mass) (Misra et al., 2012a; Ma et al., 2014; Thit et al., 2017a; Thit et al., 2017b), dissolution rate depends on the surrounding environment. For instance, decreased pH increases the dissolution of CuO NPs (Thit et al., 2017a) and available ligands present may either enhance or decrease dissolution. For instance, adsorption of ligands, such as organic acids, can affect dissolution directly or indirectly by influencing aggregation/agglomeration, which decreases surface area and thus dissolution (Mudunkotuwa et al., 2012). In addition, Cu has a high affinity for sulfur and will likely bind to inorganic sulfur in sediment. Ma et al. (2014) have reported increased dissolution after sulfidation of CuO NPs. However, the sulfidation process may also lead to the formation of a relatively insoluble metal-sulfide shell that alters surface charge and increases aggregation/agglomeration (Lowry et al., 2012; Ma et al., 2014), which may likely lead to decreased Cu bioavailability (Wang et al., 2013). Thus, copper sulfide is expected to be the predominant form of Cu in sediment under the sulfate-reducing conditions that have likely existed during the 2 years of aging (without stirring or aeration) in the present study. These conditions are also likely to occur in the aquatic environment and will be predominant in, for instance, sewer pipes and wastewater treatment plants (Ma et al., 2014). In addition, the relative distribution of CuO NPs between sediment grains and pore waters will likely change during aging (Cross et al., 2015). It was beyond the scope of the present study to characterize ^65^CuO NPs in sediments, but measurements of ^65^Cu distribution between sediment and overlying water revealed that ^65^Cu concentration in overlying water decreased and sediment concentrations increased slightly during 2 years of aging. This may indicate that the distribution of Cu changes to increase adsorption to sediment grains and lessen concentrations in pore water and overlying water.

The high background Cu concentrations in sediments and *T. tubifex* tissue (~ 30 µg Cu g^−1^ dw tissue) highlight the importance of a tracer when using a relatively low environmentally realistic exposure concentration of omnipresent metals such as Cu. Enriched stable isotope tracers make it possible to distinguish background Cu from newly accumulated ^65^Cu. Thus, it allows the detection of ^65^Cu body burdens in minute tissue samples, such as from *T. tubifex* (a few mg), despite the high Cu background levels in their tissue. One of the many advantages of this particular labeling method is that the label is incorporated into the NP during synthesis to have a unique isotopic composition throughout (Zhang et al., 2019). This makes the label more robust and reliable than labels attached to the surface, such as fluorescent markers, which can possibly be released (Schür et al., 2019). An array of papers can be consulted for further discussion on the technique (Croteau et al., 2011b; Dybowska et al., 2011; Misra et al., 2012b; Zhang et al., 2019).

### Bioaccumulation: Influence of ^65^Cu Form and Aging on *T. tubifex* Weight-Specific Body Burden

The findings on similar ^65^Cu WS-BB following exposure to the two different ^65^Cu forms are well in line with the published literature on sediment exposures of sediment-dwelling organisms to CuO NPs and dissolved Cu (Thit et al., 2015a; Thit et al., 2016; Thit and Selck, 2021). In the present study, the increases in ^65^Cu WS-BB are likely related to both water and sediment exposures. Concentrations in sediment were considerably higher than those in overlying water and may likely have contributed considerably to ^65^Cu uptake during exposures, as previously forecasted using the biodynamic model based on experiments using the same species and ^65^CuO NPs (Thit and Selck, 2021) and another oligochaete (Ramskov et al., 2015).

The lower *T. tubifex* WS-BB of ^65^Cu after 2 years of sediment aging found in the present study indicates that bioavailability decreases with prolonged aging. Although it has been suggested that transformations, such as sulfidation, may likely decrease bioavailability in sediment (Wang et al., 2013), there is no consensus on whether aged NPs are more or less bioavailable and toxic than pristine particles in sediment (Ma et al., 2014). However, soil research is well in line with the findings in the present study and have reported that bioaccumulation (Tatsi et al., 2018) and toxicity (Jośko et al., 2016) decrease with prolonged aging/contact time between NPs and soil (Jośko and Oleszczuk, 2013; Jośko et al., 2016; Tatsi et al., 2018).

### Adverse Effects

The limited adverse effects observed for either of the two ^65^Cu forms found in the present study may likely be a result of using relatively low, environmentally realistic exposure concentrations (Thit and Selck, 2021), the high tolerance of *T. tubifex* that makes this organism especially suitable for bioaccumulation studies, and high variation in some of the tested endpoints. The experiment was designed to elucidate bioaccumulation of the two ^65^Cu forms during the 28-day exposure and the influence of aging. The design proved very useful for this purpose.

*T. tubifex* survival was generally high in all treatments (>80%) during exposures to both treatments and in both freshly spiked and aged sediments. There was a slight tendency of decreased survival with increasing ^65^CuCl_2_ concentrations in both experiments, but the changes were not sufficient to eliminate that this is coincidental. Thus, no considerable differences were observed between the two ^65^Cu treatments or between the two experiments. The absence of adverse effects is similar to the findings of a previously published study (Thit and Selck, 2021), where no effects on *T. tubifex* survival or burrowing were observed*. T. tubifex* burrowing data showed high variation and were, in general, a difficult endpoint to assess in setups with four individuals per exposure container. This endpoint has previously been reported as an especially environmentally relevant endpoint (Thit et al., 2015a; Thit et al., 2020). Furthermore, CuO NPs have previously been reported to affect the burrowing behavior of sediment-dwelling species (Buffet et al., 2011) and we suggest investigating this endpoint further in the future, e.g., by collecting and weighing the fecal matter produced at the end of the exposure.

Both Cu and CuO NPs have previously been reported to affect the feeding behavior of *T. tubifex* (Thit et al., 2020; Thit and Selck, 2021) and other sediment-dwelling organisms (Ramskov et al., 2015) and is a highly environmentally relevant endpoint. However, in the present study, we observed limited effects of ^65^Cu treatment on *T. tubifex* feeding (slightly higher for controls than worms exposed to ^65^CuCl_2_ during exposures in freshly spiked sediment). Aging also only slightly affected feeding (at one concentration for each of the two ^65^Cu forms). Though WS-GR of control worms did not differ between experiments with freshly spiked sediment and aged sediment, interestingly, aging increased WS-GR of exposed worms in both ^65^Cu treatments, likely as a result of their slightly smaller size. Further investigation into the influence of aging on this endpoint is warranted.

The number of cocoons produced during 28 days was about 15 per sample (4 per worm) in all treatments and is in line with values of 5–18 cocoons per worm over a period of 72 days, reported in the literature (Kaster, 1980). CuO NPs have previously been reported to increase the hatching of zebrafish eggs (Thit et al., 2017b). Similarly, a tendency of increased hatching efficiency with increasing exposure concentration was observed in both ^65^Cu treatments. While impaired reproduction has significant implications for populations in the environment, it was very challenging practically and very time-consuming to examine reproduction in the present setup. For future studies, we suggest examining reproduction in smaller-scale experiments keeping a minimum of 10 replicates (and suggest using more due to the high variability in this endpoint) and using a lower number of treatments and fewer endpoints.

## Conclusion

The use of stable isotopically labeled ^65^Cu made it possible to detect ^65^Cu accumulation in minute tissue samples following exposure at environmentally realistic concentrations. Both ^65^CuO NPs and dissolved ^65^Cu sediment exposures resulted in increased ^65^Cu WS-BB in the freshwater oligochaete *T. tubifex*. The WS-BBs of ^65^Cu were lower in organisms exposed to sediments that were aged for 2 years than immediately after the initial spiking with ^65^CuCl_2_ and ^65^CuO NP. This indicates that transformations in sediment decrease the bioavailability of ^65^Cu in sediment. Our findings did not reveal whether prolonged aging affects the toxicity of ^65^CuO NPs in sediments. However, the decrease in organism uptake of ^65^Cu in the experiments with aged sediments suggests that a decrease in toxicity is likely to occur. These findings may aid in the understanding of bioaccumulation behavior and toxicity of metal NPs under environmentally realistic conditions. Realistic exposure concentrations and aging of NPs should preferably be included in future studies to increase environmental realism to predict the environmental risk of metal NPs accurately.

## Data Availability

The raw data supporting the conclusions of this article will be made available by the authors, without undue reservation.
