# Supplementary material for: Influence of Aging on Bioaccumulation and Toxicity of Copper Oxide Nanoparticles and Dissolved Copper in the Sediment-Dwelling Oligochaete Tubifex tubifex: A Long-Term Study Using a Stable Copper Isotope
Source: Front Toxicol. 2021 Oct 1;3:737158. doi: 10.3389/ftox.2021.737158 (PMC8915916; doi:10.3389/ftox.2021.737158)
Supplement: Supplementary file 1 [file DataSheet1.docx]

Supplementary Material

# Supplementary information: Statistics on sediment and water concentrations

## Cu exposure concentrations in sediment

### Freshly spiked sediment

For ^65^CuCl_2_ sediment ^65^Cu concentrations differed significantly among treatments (*p=* 0.001; Kruskal-Wallis) and concentrations in all spiked sediments where significantly higher than control (all p-values ≤ 0.001 for comparison between 18, 53, 112, 227 µg ^65^Cu g dw sediment and control; Conover-Inman Test). There was no significant difference between ^65^Cu concentrations in a given treatments at exposure initiation (spiked sediment) and after termination of the exposures (T_end_) (*p*-values of 0.842, 0.550, 0.324, 0.842 and 0.842, for comparison between ^65^Cu sediment concentrations after spiking and at the end of exposure for 0, 18, 53, 112, 227 µg ^65^Cu g^-1^ dw sediment, respectively), thus there were no significantly changes in exposure concentration over time.

For ^65^CuO NPs, newly added ^65^Cu concentrations differed significantly among treatments (*p*= 0.001; Kruskal-Wallis) and concentrations in all spiked sediments where significantly higher than control (all p-values ≤ 0.001 for comparison between 3, 9, 18, 39 and control; Conover-Inman Test). There was no significant difference between newly added ^65^Cu concentrations in a given treatments at exposure initiation (spiked sediment) and after termination of the exposures (T_end_) expect at 18 µg ^65^Cu g^-1^ dw sediment (*p*-values of 0.798, 0.446, 0.031, 0.099, 0.261 for comparison between ^65^Cu sediment concentrations after spiking and at the end of exposure for 0, 3, 18 and 39 µg ^65^Cu g^-1^ dw sediment, respectively), thus for most treatments, there were no significantly changes in exposure concentration over time.

### Aged sediment

For aged sediment with ^65^CuCl_2_ sediment ^65^Cu concentrations differed significantly among treatments (*p=* 0.006; Kruskal-Wallis) and concentrations in all sediments at T_0_ where significantly higher than control (all p-values ≤ 0.003 for comparison between 18, 53, 112, 227 µg ^65^Cu g dw sediment and control; Conover-Inman Test). There was no significant difference between ^65^Cu concentrations in a given treatments at exposure initiation (T_0_) and after termination of the exposures (T_end_) for all concentrations (*p*-values of 0.331, 0.623, 0.246, 0.152, for comparison between ^65^Cu sediment concentrations after spiking and at the end of exposure for 0, 18, 53, 227 µg ^65^Cu g^-1^ dw sediment, respectively; Conover-Inman), except at 112 µg ^65^Cu g^-1^ dw sediment (*p* = 0.035).

For aged sediment with ^65^CuO NP sediment ^65^Cu concentrations differed significantly among treatments (*p =* 0.002; Kruskal-Wallis) and concentrations in all sediment treatments at where significantly higher than control at beginning of exposure (T_0_) (all p-values ≤ 0.001 for comparison between 18, 53, 112, 227 µg ^65^Cu g dw sediment and control; Conover-Inman Test). There was no significant difference between ^65^Cu concentrations in a given treatments at exposure initiation (T_0_) and after termination of the exposures (T_end_) (*p*-values of 0.798, 0.446, 0.099 and 0.261, for comparison between ^65^Cu sediment concentrations after initiation and at the end of exposure for 0, 18, 112, 227 µg ^65^Cu g^-1^ dw sediment, respectively) except at 53 µg ^65^Cu g^-1^ dw sediment (*p* = 0.031). Thus there very limited significant changes in exposure concentration over the duration of exposure.

### **Freshly spiked vs aged sediment**

There was no significant difference between ^65^Cu levels in the beginning of exposures (T_0_) with newly spiked and aged sediment in control sediment (*p* = 0.694 and 0.648 for ^65^ CuCl_2_ and ^65^CuO NP, respectively). Generally, ^65^Cu concentrations in spiked sediments were slightly higher after aging than right after spiking. However the change was not significant for ^65^CuCl_2_ at 36.5 µg ^65^Cu g^-1^ dw sediment, or ^65^CuO NPs at 3, 8.9, 36.5 µg ^65^Cu g^-1^ dw sediment. The changes was however, significant for the rest of the exposure concentrations (*p*-values < 0.05).

## Cu concentrations in overlying water

In freshly spiked sediment, all ^65^Cu concentrations significantly different from each other for both ^65^CuCl_2_ treatments (all *p*-values for <0.001; Conover-Inman) and for ^65^CuO NPs (all *p*-values ≤ 0.002; Conover-Inman). In experiments with aged sediment, overlying water ^65^Cu concentrations differed significantly from each other among all treatments with ^65^CuCl_2_ (all *p*-values < 0.01) and for CuO NPs all significantly differed from control (all *p*-values < 0.01; Conover-Inman).

# Supplementary Figures

**
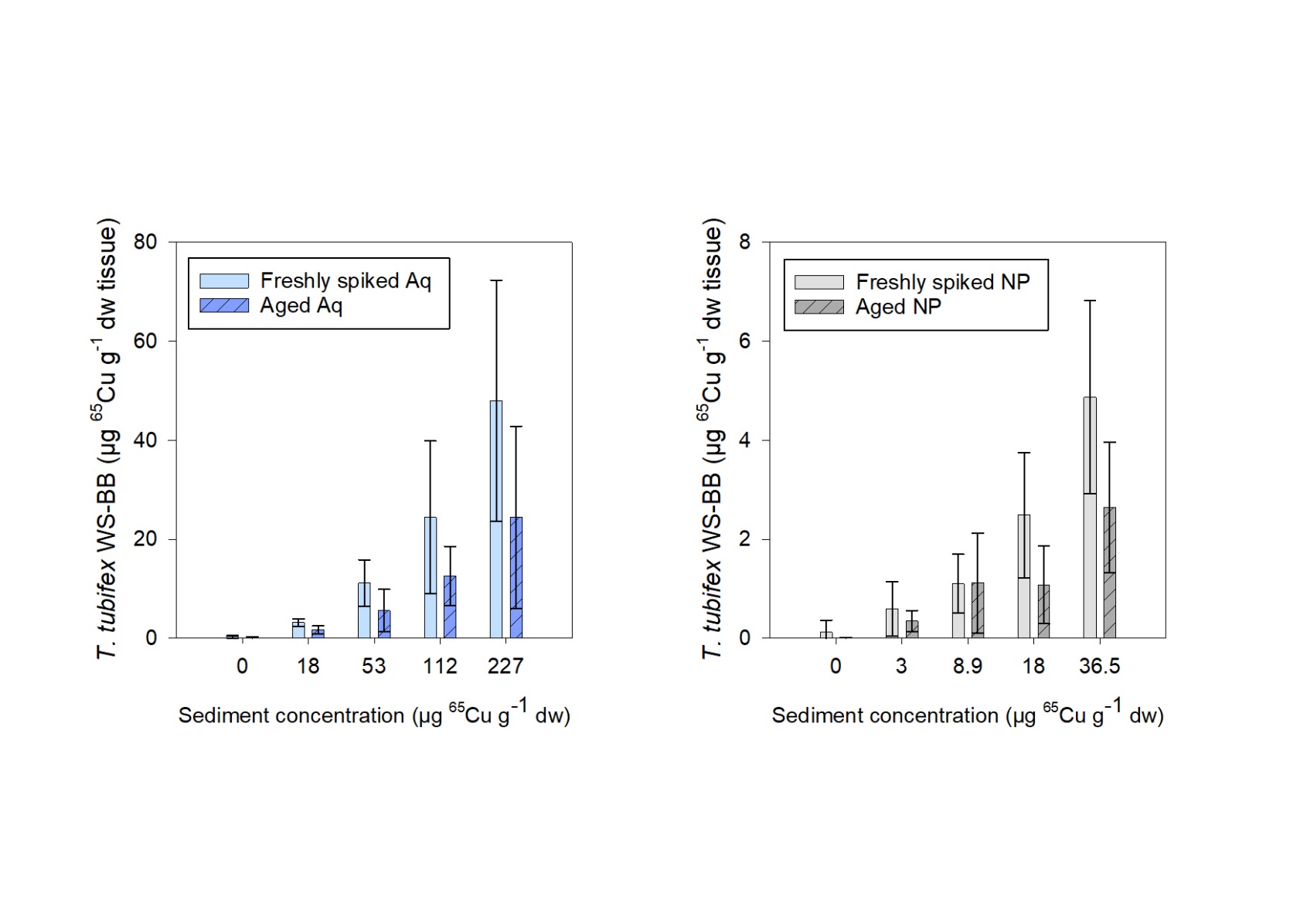
**

**Supplementary Figure 1 (S1).** WS-BB of newly accumulated ^65^Cu in *T. tubifex* after 28 day exposure to uncontaminated sediment or ^65^CuCl_2_ or ^65^CuO NPs in freshly spiked or aged sediment at 4 different concentrations.


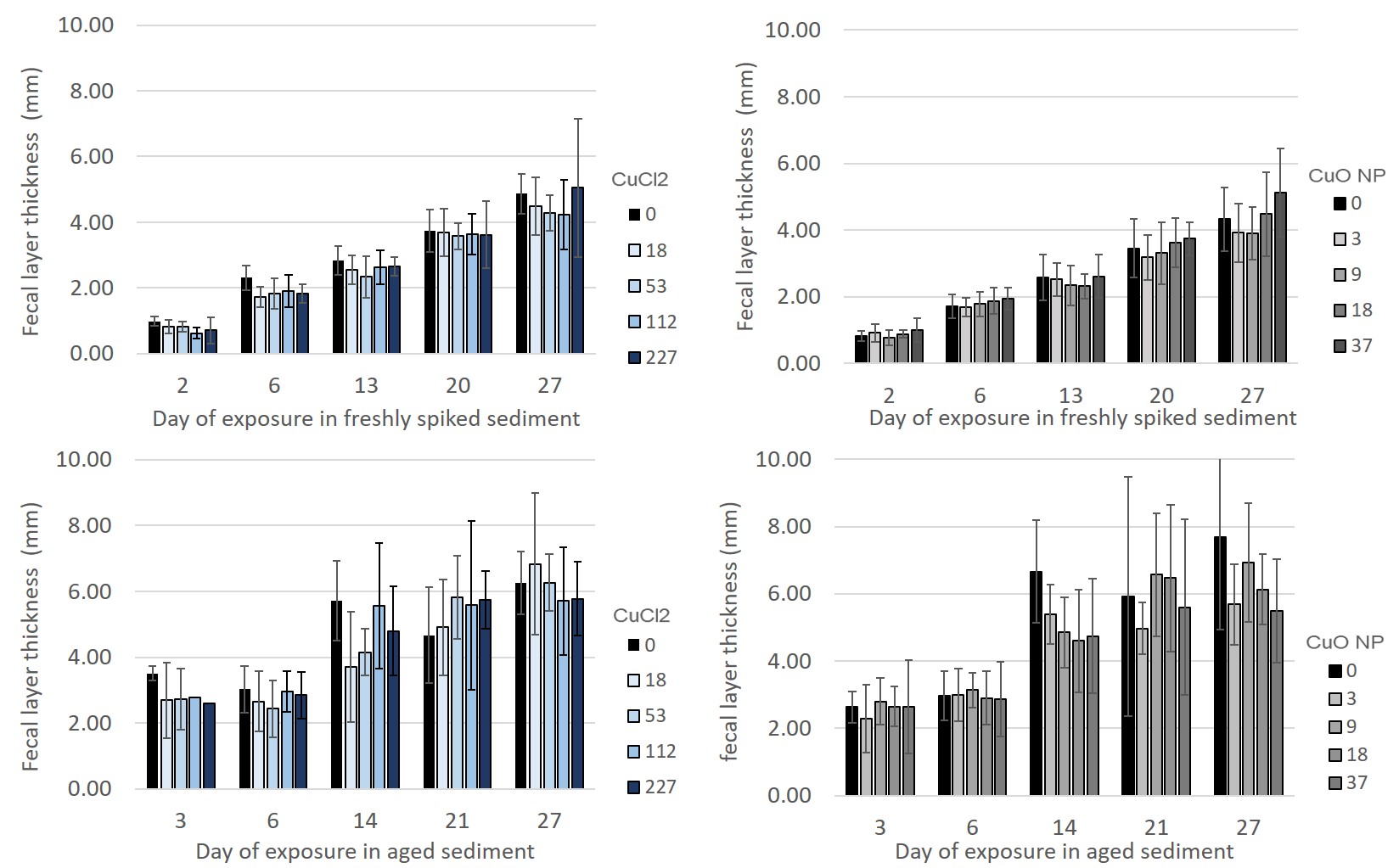


**Supplementary Figure 2 (S2).** *T. tubifex* fecal layer thickness (a proxy for feeding rate, mean ± SD, n=10) during exposures in freshly spiked (top) or aged (bottom) sediment with ^65^CuCl_2_ (left) or ^65^CuO NPs (right). Fecal layer thickness was measured with calipers (at a pre-destined place of the exposure vial).
